# Toothbrush contamination by toilet plumes: A comparative study in Chennai, India

**DOI:** 10.6026/973206300210937

**Published:** 2025-05-31

**Authors:** K Lakshmi Priya, Harikrishnan Karuppaiah, Jaideep Mahendra, Krishnarekha Kumar, Sandeep P.R., Ambalavanan Namasivayam, Uma Subbiah

**Affiliations:** 1 Department of Periodontics, Meenakshi Ammal Dental College and Hospital, Chennai, MAHER, Tamil Nadu, India

**Keywords:** Toothbrush contamination, toothbrush care, oral hygiene, hygiene practices

## Abstract

Oral health is vital to overall wellness, yet toothbrushes can harbor harmful microorganisms, especially in compromised individuals. Contamination occurs when microbes survive on surfaces such as "toilet plume" where bio-aerosols are released during flushing. Therefore, it is of interest to assess the microbial contamination of toothbrushes with and without lids, stored in different environments over a period of two months. Hence, we used data from equal student groups who used toothbrushes stored inside or outside bathrooms for this study. Samples from 36 used brushes were tested for *Candida albicans*, *Escherichia coli*, *Pseudomonas aeruginosa* and *Enterococcus faecalis* using nutrient agar, MacConkey agar and biochemical tests like IMViC and TSI. Results confirmed all four pathogens across samples, with *Candida* identified by budding yeast appearance and *Enterococcus* by a negative catalase test. These findings show that toothbrush bristles support microbial survival, which can negatively impact oral and systemic health. Thus, proper storage and regular disinfection are essential to reduce infection risk so as to maintain oral hygiene.

## Background:

Oral health is a pivotal element in bringing overall robustness [1]. Hence, managing good oral health is important for more than just maintaining the condition of our teeth [2]. It serves as a foundation for the whole wellness and well-being of our body. Therefore, being able to improve oral health may have significant systemic implications for the body, for the avoidance of diseases and consequently for the community, as well as for people's quality of life [3]. Oral diseases can be controlled by lowering the microbial load in the mouth which can be done by adopting good oral hygiene practices [1]. It is the practice of maintaining the oral cavity by thorough brushing and flossing. It has been demonstrated that maintaining good oral hygiene significantly lowers the risk of oral diseases [4]. A transition happened during World War I when hog and bone bristles were in low supply. Because China and Russia were prohibited from exporting premium natural hog bristles during World War II, nylon bristles were used in their place [5]. Because dental plaque is the key etiological component in the onset and progression of both caries and periodontal disease, effective plaque control is essential to maintain oral health. To remove plaque, which is a crucial component of disease prevention, the toothbrush is the most common tool used today [6]. Hence maintaining the toothbrush for effectively providing oral hygiene becomes essential. Unfortunately, proper toothbrush care is usually disregarded. Whilst toothbrushes are an essential component of dental care, residues on their bristles may encourage the growth of certain microorganisms. They are frequently kept in unsanitary places, including bathrooms where they are often stored. Millions of different harmful microorganisms are found in these dirty environments [7]. The retention and survival of pathogenic organisms on inanimate or biological items are referred to as contamination [8]. Additionally, bioaerosols produced by contaminated environments have the potential to contaminate surfaces. Toilet flushing aerosolizes faeces from the movement of toilet water during a flushing event (*i.e.*, bubbling, swirling, splashing). Following flushing, remaining microorganisms on the toilet walls could be released into the air later [9]. Toothbrush storage containers also have an impact on bacterial survival [9]. Numerous studies have shown that aerosols from toilet flushes can contaminate surfaces close by, including floors, toilet seats and lids. The pathogen's capacity to persist on a surface will be a key factor in determining the infection risk that it poses. *Shigella*, *E. coli*, *C. difficile*, the coronavirus that causes severe acute respiratory syndrome (SARS) and norovirus are just a few of the infections that can persist for weeks or even months on surfaces. Additionally, these bacteria may be found in an infected person's stool or vomit [10]. Prolonged toothbrush utilization encourages contamination by a variety of bacteria, including *Candida*, Lactobacilli, *Lactobacillus*, *Pseudomonas*, *Klebsiella* and *Streptococcus*. These microbes are suspected of causing infective endocarditis, gingivitis, stomatitis and dental cavities [11]. Toothbrushes are frequently only rinsed in plain water after use and kept in bathrooms or bathrooms with toilets. Exposure to the outside environment and contact with other toothbrushes also provides a perfect environment for millions of germs to thrive. These microbes require warm, moist environments to develop and thrive. It is crucial to educate the public on the proper use, maintenance and disposal of toothbrushes [12]. Therefore, it is of interest to assess the toilet plumes (bio-aerosols) contamination of toothbrushes with and without lids for various microorganisms stored in different environmental settings.

## Aims:

The aim of the study is we would like to assess the toilet plumes (bioaerosols) contamination of toothbrushes with and without lids for various microorganisms stored in different environmental settings.

## Objectives:

Toothbrush storage containers affect bacterial survival and toothbrushes may become contaminated when they come into contact with the environment. Despite the fact that there are millions of toothbrushes marketed worldwide, there seems to be little public knowledge of proper toothbrush storage. We want to emphasize the fact that using a contaminated toothbrush to clean the oral cavity can do more harm than good and may be the cause of many oral diseases.

[1] To assess the microbiological contamination on the toothbrush head and bristles with and without lids stored in bathrooms with and without attached toilets.

[2] To compare the microbiological contamination on the toothbrush head and bristles with and without lids this was stored in bathrooms with and without attached toilets.

## Materials and Methods:

This was a prospective study, conducted for a period of 2 months in Department of Periodontology in a private dental college, Chennai for a period of two months from August to October 2022. Institutional Ethics Committee approval was obtained before conducting the study. The sample size was calculated and around 36, undergraduate dental students were selected. The informed Consent form was signed by the participants prior to the study. The selected subjects were divided into four groups based on inclusion and exclusion criteria.

## Inclusion criteria:

[1] Subjects willing to participate in the study.

[2] Subjects within the age group 19 to 25 years.

[3] Should have ≥ 10 natural teeth.

[4] Clinically healthy periodontium, PPD ≤ 4 mm, with no evidence of attachment loss and bone loss on radiographs and no systemic disease

## Exclusion criteria:

[1] Aggressive periodontitis.

[2] Smoking habit.

[3] Those who had any course of medication.

## Groups:

Group I: 9 Subjects who kept toothbrush with lids inside bathrooms with an attached toilet.

Group II: 9 Subjects who kept toothbrush without lids inside bathrooms with an attached toilet.

Group III: 9 Subjects who kept toothbrush with lids outside bathrooms.

Group IV: 9 Subjects who kept toothbrush without lids outside bathrooms.

Oral hygiene instructions were explained to all study participants and oral hygiene kits including toothbrush with and without lids was provided along with common toothpaste. The number of students in each group was equally distributed for usage of toothbrushes with and without lid and also assigned to respective groups in terms of storage inside or outside the bathrooms with attached toilets by simple randomization method. After trial period, the toothbrushes were collected and were dried naturally; all 36 tooth brushes were collected in a plastic sterile container. The containers were labelled accordingly and microbiological analysis was done by Department of Microbiology in a private dental college, Chennai. The samples from the used toothbrushes (with and without lids and stores inside or outside the bathrooms with attached toilets) was analysed for four important pathogens - *Candida species*, Gram negative *Escherichia coli*, Gram negative *Pseudomonas aeruginosa* and Gram-positive *Enterococcus faecalis*. A streak of deposit between the bristle tufts was taken with a sterile cotton swab for microbial analysis (Figure 1 see PDF). Nutrient agar was used to analyse the presence of *Escherichia coli*, *Pseudomonas aeruginosa* and MacConkey agar media was used for growth of *Enterococcus faecalis*. Sabourauds Dextrose Agar was used for the growth of *Candida species* (Figure 2 see PDF). The cotton swab was then gently rubbed across the agar plate not so hard that can tear the agar plate (Figure 3 see PDF). Then streaking was done by inoculation loop. First, the flame was used to sterilize the inoculation loop. To "thin out" the germs, the inoculum was strewn around the surface of the agar (Figure 4 see PDF). The samples, was then incubated at 37°C for 24 hrs. After 24 hrs the colony growth was visible (Figure 5 see PDF).

Bacterial identification was made possible by using stains on a glass slide. Gram staining was performed wherein first the primary stain used was methyl violet and after 1 minute the glass slide was washed following which Gram's iodine (Mordant) was used. The decolorization was done using acetone for 2-3 seconds and counter stain was added with Dilute Carbol Fuchsin for 1 minute. The glass slides were allowed to dry (Figure 6 see PDF). The slides were then viewed through the microscope for identification of bacteria at 100X by oil immersion (Figure 7, 8 and 9 see PDF). The presence of *Candida albicans* in the sample was confirmed by gram staining and by the presence of budding yeast like appearance when viewed through the microscope (Figure 8 see PDF). IMViC (Indole, Methyl Red, Voges Proskauer and Citrate) was done for the positive identification for the presence of Gram-negative *Escherichia coli* and Gram- negative *Pseudomonas aeruginosa* (Figure 10 see PDF). The IMViC tests are a collection of distinct assays used in microbiology labs where the principle is to detect the ability of an organism to produce and maintain stable acid end products from glucose fermentation. Some bacteria produce large amounts of acids from glucose fermentation that they overcome the buffering action of the system (6 in proposal ref). The presence of the test microorganism *Pseudomonas aeruginosa* was confirmed by the triple sugar iron (TSI) test, citrate positive IMViC test, oxidase positive IMViC test (Figure 11, 12, 13 see PDF). Bile esculin agar (BEA) for *Enterococcus faecalis* was carried out to confirm the identification of our test microorganisms in the sample (Figure 14 see PDF). Bile esculin agar is a selective differential agar used to identify members of the *Enterococcus genus*, which was previously identified as "group D *streptococci*". Enterococci and group D *streptococci* both have the ability to hydrolyse esculin in the presence of bile (7 in proposal ref). Catalase test was negative confirming the presence of *Enterococcus faecalis* in the sample (Figure 15 see PDF).

## Results:

The purpose of the study was to emphasize the fact that using a contaminated toothbrush to clean the oral cavity can do more harm than good and may be the cause of many oral diseases. A total of 36 subjects were recruited from Department of Periodontology, in a private college, Chennai. The selected subjects were divided into the following 4 groups based on inclusion and exclusion criteria. Following the trial period, the toothbrushes were collected and microbiological analysis was done. The results were tabulated and statistical analysis was done as follows. In Group I out of 9 (100%) toothbrushes with lids kept inside bathrooms with an attached toilet, 2 (22.2%) were contaminated with *Enterococcus faecalis* and the remaining 7 (77.8%) were not contaminated. In Group II out of 9 (100%) toothbrushes without lids kept inside bathrooms with an attached toilet, 5 (55.6%) were contaminated with *Enterococcus* and the remaining 4 (44.4%) were not contaminated. In Group III and Group IV none of the toothbrushes with or without lids which were kept outside the bathrooms were contaminated with *Enterococcus* (Figure 16 see PDF). So, it was understood that *Enterococcus* contamination was higher when toothbrushes were placed without lids and inside bathrooms with an attached toilet. This difference was found to be statistically significant (p=0.008) (Table 1). In Group I and Group II None of the toothbrushes with or without lids which were kept inside the bathrooms with an attached toilet were contaminated with *Pseudomonas aeruginosa*. In Group III out of 9 (100%) toothbrushes with lids kept outside bathrooms, 4 (44.4%) were contaminated with *Pseudomonas aeruginosa* and the remaining 5 (55.6%) were not contaminated. In Group IV out of 9 (100%) toothbrushes without lids kept outside bathrooms, 5 (55.6%) were contaminated with *Pseudomonas aeruginosa* and the remaining 4 (44.4%) were not contaminated (Figure 17 see PDF). So, it was understood that *Pseudomonas aeruginosa* contamination was higher when toothbrushes were placed without lids and outside bathrooms. This difference was found to be statistically significant (p=0.006) (Table 2). In Group I out of 9 (100%) toothbrushes with lids kept inside bathrooms with an attached toilet, 4 (44.4%) were contaminated with *Candida albicans* and the remaining 5 (55.6%) were not contaminated. In group II None of the toothbrushes without lids which were kept inside the bathrooms with an attached toilet were contaminated with *Candida albicans*. In Group III out of 9 (100%) toothbrushes with lids kept outside bathrooms, 4 (44.4%) were contaminated with *Candida albicans* and the remaining 5 (55.6%) were not contaminated. In Group IV out of 9 (100%) toothbrushes without lids kept outside bathrooms, 5 (55.6%) were contaminated with *Candida albicans* and the remaining 4 (44.4%) were not contaminated (Figure 18 see PDF). However, this difference between four study groups with regard to toothbrush contamination with *Candida albicans* was not statistically significant (p>0.05) (Table 3). In all the 4 Groups none of the toothbrushes were contaminated with *Escherichia coli* (Table 4) (Figure 19 see PDF). In a total of 36 toothbrushes 7 (19.4%) were contaminated with *Enterococcus faecalis* and the remaining 29 (80.6%) were not contaminated. In a total of 36 toothbrushes 9 (25.0 %) were contaminated with *Pseudomonas aeruginosa* and the remaining 27 (75.0 %) were not contaminated. In a total of 36 toothbrushes 13 (36.1 %) were contaminated with *Candida albicans* and the remaining 23 (63.9%) were not contaminated. In a total of 36 toothbrushes none of the samples were contaminated with *Escherichia coli* (Table 5), (Figure 20 - see PDF).

## Discussion:

Brushes have been highlighted as the most popular oral hygiene tool to promote oral health and prevent dental illnesses. This is because of the growing knowledge of the importance of maintenance of oral health and dental professional's emphasis on preventive measures [11]. The retention of germs is greatly aided by toothbrushes. According to Svanberg (1978) toothbrushes can harbour a lot of bacteria. Toothbrushes can become contaminated through environmental contact and brush storage containers also fairly contribute on bacterial survival [13]. According to Cobb *et al.* 1920 a toothbrush is a significant contributor to recurrent oral infections [14]. An oral cavity recontamination risk is posed by the colonisation of harmful microorganisms on toothbrushes while being stored in unclean settings [7]. For bacteria to survive, storage conditions for toothbrushes are crucial. According to Dayoub *et al.* 1977 [15] and Meier *et al.* 1996, toothbrushes stored in aerated settings had less microorganisms than those kept in carrier containers [16]. University of Alabama researchers discovered that brushes kept in the bathroom are very likely to have faecal matter present in the bristles. It has been demonstrated that toilet flushing creates an aerosol spray of contaminated water that can contaminate the bristles of the tooth brush [17]. In Group 1 for subjects who kept the brush with lids inside bathroom showed the presence of *Enterococcus faecalis* and **Candida* albicans* this could be because the toothbrushes were kept in enclosed areas, aerosols from the restroom can contaminate the brushes with millions of microorganisms and enteric bacteria. This was in accordance to Peker et al study in 2015 where the study subjects kept their toothbrushes inside of sealed containers to prevent contamination from the outside environment [18]. The findings were consistent with the following studies as well wherein Dayoub *et al.* discovered that compared to toothbrushes left open to the air, those kept in closed containers and exposed to contaminated surfaces produced high bacterial counts. It is shown that a cap to store a toothbrush improved microbial contamination. It is further shown that more environmental humidity more the survival of germs on toothbrushes and germs may survive in dampness for longer than 24 hours [19].

In Group II for subjects who kept the brush without lids inside bathroom showed the presence of *Enterococcus faecalis*. The presence of *Enterococcus faecalis* in toothbrush is a sign of potential faecal contamination. It's likely that the used toothbrushes were kept in unsanitary locations like bathroom sinks and toilets. The humid climate in the bathroom promotes the development of this microorganism. This hazard could be linked with "toilet plume" bioaerosols created by flushing toilets. The small particles that are left over after a droplet's water evaporation are called droplet nuclei. They float with the currents of the air naturally because they have a very low settling velocity. Jessen noticed that when flush energy grew, the amount of bioaerosol also did. [10]. Studies have shown that after rinsing a used toothbrush in plain water, people typically store it in the bathroom or toilet. Releasing aerosols from flushing toilets, contaminated hands, commensals and *Pseudomonas* from the bathroom and other wet areas, contaminate the tooth brushes, this practise promotes the growth of various germs and the spread of enteric bacteria [11]. The present study's findings proved that the area where tooth brushes are kept after use might serve as a cause of bacterial contamination stressing the value of proper care. For Group III the brushes kept outside bathrooms with lids showed the presence of both *Pseudomonas aeruginosa* and *Candida albicans*. Due to the fact that *Pseudomonas aeruginosa* is common in nature, including water, the bacteria may have also entered into the toothbrushes through the rinsing water. By using tap water to rinse toothbrushes, According to a study, using a cap over the toothbrush head encouraged the growth of germs, which prefer a moist environment especially *Candida species*.

For Group IV the brushes kept outside bathrooms without lids showed the presence of both *Pseudomonas aeruginosa* and *Candida albicans*. According, to Dayoub *et al.* (1977) after use, the number of bacteria on toothbrushes stored in room temperature drops more quickly than those kept in containers. According to Bunetel *et al.* research, microorganisms become trapped inside the toothbrush's bristles and their ability to survive depends on their kind (aerobic versus anaerobic) and toothbrush design and also the environment they are stored [20]. The present study's finding was also consistent with study done by Sowmiya *et al.* wherein it was stated that Long-term toothbrush use encourages contamination by a variety of bacteria, including *Candida*, *Streptococcus*, *Lactobacilli*, *Pseudomonas*, *Klebsiella* and *Escherichia coli*. Contact with other toothbrushes and exposure to the outside environment as the toothbrushes were exposed directly to the environment and not covered by cap were considered the source of contamination [11]. In the present study none of the four groups showed contamination of *Escherichia coli*. This could be due to *E. coli* is seen only in gut an enteric flora which is a normal commensal and so the possibility for the toothbrushes to be contaminated through aerosol or toilet plumes when placed inside the bathroom with and without lids does not justify. Moreover, even if they are present in the aerosol the *E. coli* bioaerosol remained airborne and viable only for 4 to 6 hours after flushing [10]. Based on the results obtained it can be suggested that it is crucial to stress that brushing the teeth with contaminated materials may cause diseases to develop and that entirely depends on the type and quantity of bacteria isolated from toothbrushes and the nature or type of the bacteria is largely influenced by the environment they are stored.

## Limitations:

Toothbrush that helps to maintain the oral health has to be maintained well and be made free of microorganism to achieve that goal. Although, the present study was done to find the bacterial contamination of 4 specific bacteria namely *Candida species*, Gram negative *Escherichia coli*, Gram negative *Pseudomonas aeruginosa* and Gram-positive *Enterococcus faecalis* on the toothbrush with lid and without lid inside and outside the restrooms it did have its limitation. We cannot draw conclusions based on the small sample size. Additionally, only the identification of the presence or absence of the microorganism was done. The colony counting was not done in the present study. The presence of toothpaste contamination between the bristle tufts and also the composition of the toothpaste also had significant effect on reducing the growth of microorganism [21].

## Conclusion:

Toothbrushes should not be stored in bathrooms, especially shared ones, as they can harbor infections. Storing multiple uncovered brushes together allows them to touch and spread germs. Regular disinfection is essential to prevent reinfection, maintain oral hygiene and support overall health. The ADA has recommended replacing toothbrushes every three months since 1996. Consistent maintenance practices are necessary to reduce contamination risks and minimize exposure to harmful bacteria, which is crucial for effective oral care.

## Figures and Tables

**Table 1:** Comparison of toothbrush contamination with *Enterococcus faecalis* among 4 study groups

**Groups**		**Enterococcus**		**p-value**
		**Absent**	**Present**		
Group I- Toothbrush with lids inside bathrooms with an attached toilet	Count	7	2	0.008
	%	77.80%	22.20%	
Group II- Toothbrush without lids inside bathrooms with an attached toilet	Count	4	5	
	%	44.40%	55.60%	
Group III- Toothbrush with lids outside bathrooms	Count	9	0	
	%	100.00%	0.00%	
Group IV- Toothbrush without lids outside bathrooms.	Count	9	0	
	%	100.00%	0.00%	

**Table 2:** Comparison of toothbrush contamination with *Pseudomonas aeruginosa* among 4 study groups

**Group**		**Pseudomonas**		**Total**	**p-value**
		**Absent**	**Present**		
Group I -Toothbrush with lids inside bathrooms with an attached toilet	Count	9	0	9	0.006
	%	100.00%	0.00%	100.00%	
Group II-Toothbrush without lids inside bathrooms with an attached toilet	Count	9	0	9	
	%	100.00%	0.00%	100.00%	
Group III-Toothbrush with lids outside bathrooms	Count	5	4	9	
	%	55.60%	44.40%	100.00%	
Group IV- Toothbrush without lids outside bathrooms	Count	4	5	9	
	%	44.40%	55.60%	100.00%	

**Table 3:** Comparison of toothbrush contamination with *Candida albicans* among 4 study groups

**Group**		***Candida albicans***		**Total**	**p-value**
		**Absent**	**Present**		
Group I- Toothbrush with lids inside bathrooms with an attached toilet	Count	5	4	9	0.069
	%	55.60%	44.40%	100.00%	
Group II-Toothbrush without lids inside bathrooms with an attached toilet	Count	9	0	9	
	%	100.00%	0.00%	100.00%	
Group III- Toothbrush with lids outside bathrooms	Count	5	4	9	
	%	55.60%	44.40%	100.00%	
Group IV- Toothbrush without lids outside bathrooms	Count	4	5	9	
	%	44.40%	55.60%	100.00%	

**Table 4:** Comparison of toothbrush contamination with *Escherichia coli* in four study groups

**Groups**		***E. coli***	**Total**
		**Absent**	
Group I- Toothbrush with lids inside bathrooms with an attached toilet	Count	9	9
	%	100.00%	100.00%
Group II- Toothbrush without lids inside bathrooms with an attached toilet	Count	9	9
	%	100.00%	100.00%
Group III- Toothbrush with lids outside bathrooms	Count	9	9
	%	100.00%	100.00%
Group IV -Toothbrush without lids outside bathrooms	Count	9	9
	%	100.00%	100.00%

**Table 5:** Total percentage of individual microorganisms in all 4 groups

**Total percentage of microorganisms**		**All 4 groups**		**p-value**
		**Absent**	**Present**	
*Enterococcus faecalis*	Count	29	7	0.008
	%	80.60%	19.40%	
*Pseudomonas aeruginosa*	Count	27	9	
	%	75.00%	25.00%	
*Candida albicans*	Count	23	13	
	%	63.90%	36.10%	
*Escherichia coli*	Count	36	0	
	%	100.00%	0	
